# Gender, Success, and Drop-Out during a Resistance Exercise Program in Community Dwelling Old Adults

**DOI:** 10.1155/2017/5841083

**Published:** 2017-08-14

**Authors:** O. G. Geirsdottir, M. Chang, K. Briem, P. V. Jonsson, I. Thorsdottir, A. Ramel

**Affiliations:** ^1^The Icelandic Gerontological Research Center, Reykjavik, Iceland; ^2^Faculty of Food Science and Nutrition, University of Iceland, Reykjavik, Iceland; ^3^Department of Sport Science, School of Science and Engineering, Reykjavik University, Reykjavik, Iceland; ^4^Department of Physiotherapy, University of Iceland, Reykjavik, Iceland; ^5^Department of Geriatrics, National University Hospital of Iceland, Reykjavik, Iceland; ^6^School of Health Sciences, University of Iceland, Reykjavik, Iceland

## Abstract

**Background:**

Resistance exercise training can be effective against sarcopenia. We identified predictors of drop-out and compared physical outcomes between men and women after such training.

**Methods:**

Subjects (*N* = 236, 73.7 ± 5.7 years) participated in a 12-week resistance exercise program. Outcome variables were measured at baseline and endpoint.

**Results:**

Drop-out was 11.9% and not significantly different between genders. Drop-outs were significantly older and had poorer strength and physical function in comparison to completers. Anthropometrics, QoL, and cognitive function were not related to drop-out. According to multivariate analysis, gait speed and physical activity were the strongest predictors of drop-out. After the training, gains in lean mass or appendicular muscle were significantly higher in men than women; however relative gains in appendicular muscle as well as absolute improvements in strength and function were similar in men and women, respectively.

**Conclusions:**

Participants who drop out are older, have poorer physical function, and are less physically active. Old women do not drop out more frequently than men and show meaningful improvements in relevant outcomes similar to men after such a training program. The trial is registered at the US National Library of Medicine (NCT01074879).

## 1. Introduction

Sarcopenia, the age related loss of muscle mass and strength, is frequently observed in older populations [1]. It is an unfavorable condition, because it is related to functional decline and adverse health outcomes [1]. Thus, it is important to establish therapies that prevent sarcopenia or delay its onset. Regular resistance training has been shown to be an effective intervention [2].

However, a resistance exercise program can be demanding for participants, because often high intensity resistance exercise is prescribed for old adults [2]. The rationale for this is that gains in muscle strength and size are thought to be larger following resistance training with higher intensities, although the degree of training-induced muscle hypertrophy is generally small in old adults [2].

During a high intensity training program, participants typically attend two to three sessions per week and a weekly increase in load is implemented in order to keep the intensity of around 80% (relative to the one-repetition-maximum) constant due to gains in strength that occur during a resistance exercise program [2, 3].

Drop-out during resistance exercise programs has been reported to be 22–38% [4–6] and increases with the length of the program [7]. In this context, it is important to identify participants at higher risk for drop-out beforehand, in order to provide these individuals with better supervision and attention during a resistance exercise program with the aim of preventing drop-out.

When it comes to compliance with resistance exercise, women might need particular attention. In general, women have been reported to have lower levels of physical activity than men [8] and this is also true for resistance exercise [9]. It has been suggested that women increase muscle mass less than men with the same amount of training [10]. A recently published study reported sex differences in the response to resistance exercise training also in old people with lesser improvement of maximal torque and muscle quality in women compared to men [10]. In addition to biological differences in muscular adaptation to physical training, one might also speculate that the gym environment itself could be a deterrent for some female participants [7].

In order to gain knowledge on drop-out and success after a resistance exercise program, we conducted this secondary data analysis based on results from a previously published randomized, controlled trial, designed to examine the effect of postexercise protein ingestion on the efficacy of strength training in old adults [3]. The aim of the present work was (1) to identify baseline predictors of drop-out with particular focus on physical function and (2) to compare changes in physical outcomes after the resistance exercise program between men and women.

## 2. Methods

Several papers have been published from this study and in the present paper we followed the methods of Geirsdottir et al. 2012 [11], Arnarson et al. 2013 [3], Arnarson et al. 2014 [12], and Ramel et al. 2015 [13]. The similarity found between the present paper and the above-mentioned articles is restricted to Methods. The present paper contains novel scientific content with focus on drop-out, success, and gender not presented previously.

### 2.1. Subjects

Participants (*N* = 236, range 65–92 years) were recruited within the Capital Area in Iceland. Exclusion criteria were major orthopedic disease, pharmacological interventions known to affect muscle mass, and low cognitive function according to Mini-Mental State Examination (MMSE) ≤ 19 points [12, 14]. Subjects had also to be free from disorders that could significantly affect their body composition. Some of the participants had hypertension, hyperlipidemia, or type 2 diabetes [15], although the enrolled subjects looked apparently healthy. The study received permission from the Icelandic National Bioethics Committee (VSNb2008060007/03-15) and has therefore been performed in accordance with the ethical standards as described by the Declaration of Helsinki 1964 and its later amendments. The subjects gave their written informed consent before participation in the study.

### 2.2. Resistance Training

The aim of the resistance exercise program was to increase strength and mass of all major muscle groups. In order to assess physiological changes, measurements were conducted at baseline and the end of the study. The training was conducted in a local fitness study where participants trained three days per week for 12 weeks in supervised groups of 26–44 individuals. Each training started with a 10–15 minute warm up. The correct exercise and lifting techniques were taught at lower loads (60% of 1-repetition maximum (1-RM)) during the first week. In the following weeks resistance training was done in three sets, where each exercise was repeated six to eight times, at 75–80% of 1-RM. After each exercise the participants rested shortly until they felt ready for the next exercise. In order to keep the number of repetitions per exercise unchanged during the 12 weeks of training, training loads were increased by 5–10% each week. Stretching exercises were performed at the end of each session. The training was supervised by staff members, by fitness trainers, and occasionally by a physiotherapist. After each training, participants received a protein and carbohydrate containing drink, which was part of the original aim of this trial [3]. As the supplementation was not related to outcomes of the present analysis, it is not considered further in this paper.

### 2.3. Body Composition

Body weight (BW) was measured in light underwear (Seca scale model number 708, Seca, Hamburg, Germany) and height was measured with a calibrated stadiometer (model number 206; Seca, Hamburg, Germany). Body mass index (BMI) was calculated as kg/m^2^. Waist circumference was measured halfway between the top of the lateral iliac crest and the lowest rib. Body composition (lean body mass = LBM, fat mass = FM, and appendicular skeletal muscle = ASM) was assessed at the Icelandic Heart Association, Kopavogur, Iceland, using dual energy X-ray absorptiometer (DXA, Hologic QDR-2000 plus®, Hologic Inc., Waltham, MA, USA).

### 2.4. Physical Function

#### 2.4.1. Six-Minute Walk for Distance (6MWD)

The 6MWD was conducted in a gym hall according to the guidelines from the American Thoracic Society [16]. In healthy subjects, the 6MWD usually ranges from 400 to 700 m [17–19]. For some of the statistical analyses, subjects were categorized into* below 400 m* and in* at least 400 m* (see Figure 2).

#### 2.4.2. Quadriceps Strength

Quadriceps strength (maximum voluntary isometric contraction (MVIC)) was tested using an isokinetic dynamometer (Kin-Com® 500H Chattanooga). The participants performed three submaximal trials and then four MVIC tests for five seconds each, with a 50-second rest between tests. The greatest output was recorded as the peak torque expressed in Newton (N).

#### 2.4.3. Timed Up and Go (TUG) Test

The participants were instructed to stand up from a chair (seat height = 43 cm), to walk 3 m, then to turn around, to return, and to sit down again. Time (sec) was taken during this procedure. The participants wore shoes during this test and used walking aids when necessary [20].

#### 2.4.4. Grip Strength

Hand grip strength (lbs) was measured using a hydraulic hand dynamometer (Baseline® Baseline Evaluations Corporation). The maximal achieved grip strength from three attempts was recorded as the subject's grip strength.

### 2.5. Questionnaires

Questionnaires were used to receive information on demographic characteristics and medication count.

### 2.6. Leisure-Time Physical Activity (LTPA)

LTPA during the last year was estimated using a questionnaire [21] based on the Compendium of Physical Activities [22] and Paffenberger's questionnaire [23]. It contains questions on the subjects' participation in sports, exercises, or other physical activities. In statistical analysis LTPA is used as min/week.

### 2.7. Health Related Quality of Life (HRQL)

The HRQL questionnaire involves 12 domains that cover a range of psychological and physical functions, for example, functional status, vitality, social function, physical pain, emotions, general health, and mental health. The domains were summarized into a single score and converted into a *T*-score corrected for age and gender (*T*-score norm is 50 ± 10) [24, 25].

### 2.8. Statistical Analysis

Statistical analysis was conducted using SPSS for Windows version 22.0 (SPSS, Chicago, IL, USA). The Kolmogorov-Smirnov test was used to investigate the distribution of the data. In Tables 1–3, data are shown as mean ± standard deviation (SD). Comparison between groups, for example, men and women, was done using independent samples' *t*-test (normally distributed variables) or Mann–Whitney *U* test (not normally distributed variables). Correlations between variables were calculated using Pearson's correlation coefficient *r*. Logistic regression models with various degrees of statistical correction were used to find out whether physical function can predictor drop-out. Model 1 is crude analysis; model 2 corrected for age; model 3 additionally corrected for gender. Risk of drop-out for categories of gait speed and five-year age groups was calculated using cross tabs including chi-square statistics. The level of significance was set at *P* < 0.05.

## 3. Results

Baseline data and differences between genders can be seen in Table 1. There were differences between men and women in body composition and muscular strength. However, physical function (6MWD, TUG) did not differ between men and women. Drop-out rate was insignificantly higher in women. In comparison to completers, participants who dropped out were older and had poorer strength and physical function. They exercised less; however, neither body composition nor QoL nor MMSE was related to drop-out (Table 2).

In general, the intervention resulted in favorable changes in body composition (FM −0.44 kg, LBM +0.75 kg, and ASM +0.53 kg, all *P* < 0.001), muscular strength (quadriceps +53 N; grip strength +3 lb, both *P* < 0.001), and physical function (6MWD +34 m; TUG −0.7 sec, both *P* < 0.001). Attendance rate was 91% and similar in both genders. Gains in LBM and ASM were not related to improvements in physical function; however, increases in quadriceps strength correlated with improvements in TUG (*r* = 0.151, *P* = 0.032) and 6MWD (*r* = 0.137, *P* = 0.052).

Changes after the intervention stratified by gender can be seen in Table 3. Although absolute gains in LBM or ASM were higher in men than women, relative gains in muscle as well as improvements in strength und function were similar between genders.

Logistic regression models in Table 4 show the prediction of drop-out by variables of physical function and physical activity. In the crude models without any statistical correction (model 1) most of the variables describing physical function and physical activity were associated with the risk of drop-out in a negative way (i.e., better function-lower risk of drop-out). However, some of these associations were partly explained by age and gender. In models 2 and 3 in which we included statistical correction for age and gender, the associations between function and drop-out got weaker and are partly no longer significant. According to models 3 physical activity per week and gait speed (borderline significant) were the strongest predictors for drop-out from all the physical function variables.

**Figure 1:**
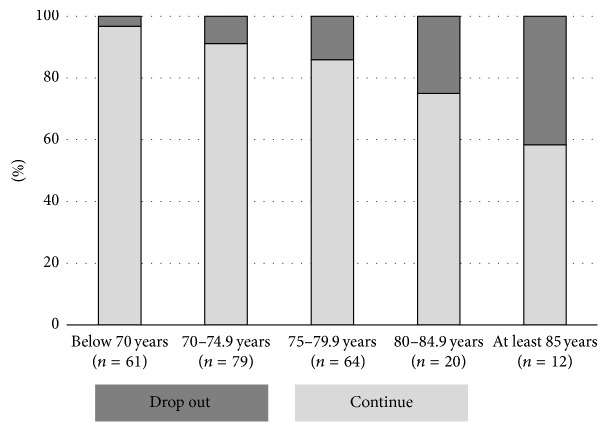
Associations between age categories and risk of drop-out (chi-square statistics, *P* < 0.001).

**Figure 2:**
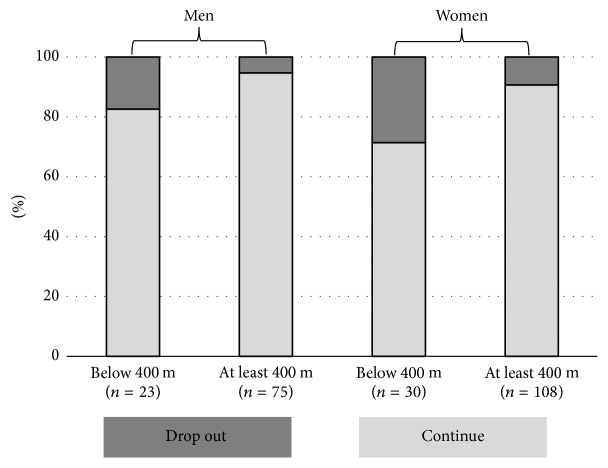
Associations between gait speed categories and risk of drop-out (chi-square statistics, *P* = 0.065 for men and 0.008 for women; *P* < 0.001 for men and women combined).

Figures 1 and 2 show the risk for drop-out depending on age groups and 6MWD, respectively. Both higher age and lower distance were associated with higher drop-out (chi-square statistics).

## 4. Discussion

The present study investigated drop-out and success of community dwelling old adults after a 12-week resistance exercise program with particular consideration of potential gender differences. In our study drop-out of 11.9% was low compared to previously reported studies [4–6] and not significantly different between genders. There are several potential reasons why drop-out was low: (1) our study population consisted of rather healthy volunteers who did neither represent the general population at this age nor a clinical sample of patients. It can be assumed that volunteers show higher motivation and compliance towards physical training. (2) Further, the study was conducted during the lively opening hours in (at that time) one of the most modern fitness studios in the Reykjavik area as opposed to a physiotherapy facility at a hospital. (3) Training was conducted in groups of 26–44 individuals with several dedicated instructors and study staff assisting and being available for help. Although not measured, we found that there was a pleasant and kind atmosphere and that participants liked to attend the training sessions which is reflected by the high attendance rate of 91%.

Drop-out was related to baseline strength, physical function, medical drug use, and physical activity but unexpectedly not to body composition, MMSE, or QoL. According to multivariate analysis age and gait speed were the strongest baseline predictors. It is a matter of definition at what percentage drop-out is still acceptable or not. However, as shown in Figures 1 and 2, age older than 80 years and 6MWD below 400 m were both related to a drop-out risk of >20%. Consequently, these cut-off values for age and gait speed can be easily used to identify participants at higher risk for drop-out. Future studies are needed to determine whether, for example, better supervision in smaller groups might help to prevent attrition from a study program in such vulnerable participants.

Considering that the oldest and weakest individuals had the highest risk of drop-out, it can be speculated that the program was too demanding for them. Although it is widely accepted that resistance exercise with high intensity (heavy loads) is efficient [26], a controversy has arisen during recent years whether strength and muscle mass gains would also be achievable with lighter load resistance exercise programs [27, 28]. There is evidence that, when matched for mechanical work, resistance exercise provokes substantial gains in muscle mass and strength, irrespective of whether heavy or rather lighter loads were used [29].

According to a recently published meta-analysis [2] on this topic, both high and moderate resistance exercise provoke significant gains in muscle strength compared with nontraining control. However, differences between high and moderate intensities are less clear, partly due to methodological issues of the included studies. An analysis of the subset of studies to report data on the training-related changes in muscle size revealed that neither the higher nor lower load resistance training programs performed were effective in inducing significant muscle hypertrophy [2]. However, this result is most likely a consequence of short intervention periods and/or lack of an appropriate surplus of calories and protein necessary for muscle gain. As long-term adherence to resistance exercise is a general problem [30], we think that minimum effective thresholds should be determined in order to increase long-term success.

It has been proposed that a short-term increase in testosterone after heavy resistance exercise promotes muscle protein synthesis, which finally leads to protein accretion and hypertrophy [31]. Women have much lower testosterone concentrations than men and do not experience postexercise increases in testosterone compared with men [32, 33]. This sex specific response has been used to explain why women have an attenuated potential for resistance exercise-induced hypertrophy [34]. In accordance with this, women gained significantly less LBM and ASM than men in our study. However, we also found that both relative gains in LBM and ASM and clinically relevant improvements in muscle strength and physical function after a resistance exercise program were similar between genders. In order to put the improvements of TUG (−0.7 sec) and 6MWD (+34 m) in perspective, it is worth considering that age related changes have been reported +1 sec/decade for TUG [35] and −30 m/decade [36] for 6MWD. Our study also indicates that gains in muscle strength seem to be more important than gains in muscle mass in order to maintain or improve physical function. Strength gains without associated alterations in body composition but better functional status as seen in the present study possibly reflect enhanced neural adaptation in the early stages of training [37].

## 5. Limitations

The participants in our study were apparently healthy and motivated community dwelling volunteers. Thus, the reported drop-out rate most likely underestimates drop-out experienced in a population representative for this age or in a clinical sample. Further, our resistance exercise intervention was twelve weeks. A longer program would have increased drop-out rate as studies have shown increased drop-out with increased study length [38, 39]. Further, our study failed to establish the reasons why subjects finally decided to drop out, as correlation does not reflect necessarily causality. This could be related to a number of factors not assessed in the present study, including greater perceived exertion during exercise, pain, or social considerations. In future studies, qualitative interviews with subjects after drop-out can help to elucidate the reason.

## 6. Conclusion

Our study shows that participants who drop out are older, have poorer physical function and poorer health, and are less physically active. Great care has to be taken of such individuals in order to help them to participate in resistance exercise. Age > 80 years and 6MWD < 400 m might be useful screening parameters to identify participants at higher risk for drop-out.

Although there a theoretical rationale that old women improve less than old men after a resistance exercise program, we found that changes in strength and function were similar between genders and reflect improvements equal to 7–11 years of younger age.

## Figures and Tables

**Table 1:** Characteristics of male and female participants.

	Male	Female	*P* value^*∗*^
	(*n* = 98)	(*n* = 138)	
	Mean ± SD	Mean ± SD	
Age (years)	74.6 ± 5.9	72.8 ± 5.5	0.018
BMI (kg/m^2^)	29.7 ± 4.6	28.1 ± 4.9	0.012
Waist (cm)	108 ± 12	94 ± 13	<0.001
Body fat (%)	33.9 ± 5.9	41.3 ± 6.5	<0.001
Body fat (kg)	32.3 ± 10.4	31.4 ± 9.5	0.515
Lean body mass (kg)	57.4 ± 7.2	41.1 ± 4.7	<0.001
Appendicular skeletal muscle (kg)	29.6 ± 3.9	20.9 ± 2.6	<0.001
Appendicular skeletal muscle (%)	31.9 ± 3.2	28.3 ± 2.9	<0.001
Quadriceps strength (N)	538 ± 124	409 ± 90	<0.001
Quadriceps strength^*∗∗*^ (N/kg)	5.9 ± 1.5	5.6 ± 1.3	0.100
Grip strength (lb.)	79 ± 15	49 ± 9	<0.001
6MWD (m)	456 ± 86	452 ± 76	0.708
TUG (sec)	8.0 ± 2.1	7.9 ± 2.3	0.595
MMSE (score)	27.1 ± 2.1	27.8 ± 2.0	0.021
QoL (score)	54.9 ± 6.2	55.0 ± 6.1	0.907
Number of drugs	2.3 ± 1.4	1.9 ± 1.6	0.067
Exercise/week (min)	323 ± 341	356 ± 343	0.472
Drop-out (%)	8.2	14.5	0.138

^*∗*^Differences between genders according to independent samples *t*-test (normally distributed variables) and Mann–Whitney *U* test (not normally distributed variables). ^*∗∗*^Corrected for body weight.

**Table 2:** Characteristics of participants that dropped out or completed the program.

	Completers	Drop-out	*P* value^*∗*^
	(*n* = 208)	(*n* = 28)	
	Mean ± SD	Mean ± SD	
Age (years)	73.0 ± 5.5	77.5 ± 5.9	<0.001
BMI (kg/m2)	28.8 ± 4.8	29.0 ± 4.9	0.853
Waist (cm)	99.7 ± 14.4	100.4 ± 14.5	0.815
Body fat (%)	37.9 ± 7.3	40.8 ± 6.8	0.073
Body fat (kg)	31.5 ± 9.6	33.6 ± 12.0	0.325
Lean body mass (kg)	48.2 ± 9.8	45.3 ± 11.1	0.175
Appendicular muscle mass (kg)	24.7 ± 5.2	22.4 ± 6.0	0.052
Appendicular skeletal muscle (%)	30.0 ± 3.5	28.2 ± 2.6	0.022
Quadriceps strength (N)	471 ± 122	386 ± 117	0.005
Quadriceps strength^*∗∗*^ (N/kg)	5.8 ± 1.4	4.9 ± 1.5	0.021
Grip strength (lb)	63 ± 19	53 ± 18	0.045
6MWD (m)	461 ± 74	394 ± 102	<0.001
TUG (sec)	7.7 ± 1.9	9.8 ± 3.7	<0.001
MMSE (score)	27.6 ± 2.0	27.0 ± 2.4	0.175
QoL (score)	55.1 ± 6.2	53.5 ± 5.1	0.199
Number of drugs for different conditions	2.0 ± 1.5	2.6 ± 1.6	0.058
Exercise/week (min)	364 ± 350	181 ± 210	<0.001

^*∗*^Differences between genders according to independent samples *t*-test (normally distributed variables) and Mann–Whitney *U* test (not normally distributed variables). ^*∗∗*^Corrected for body weight.

**Table 3:** Changes in anthropometric, physical, and functional outcomes in male and female participants after the 12-week intervention.

	Male	Female	*P* value^*∗*^
	(*n* = 91)	(*n* = 119)	
	Mean ± SD	Mean ± SD	
BMI (kg/m^2^)	0.21 ± 0.58	0.09 ± 0.52	0.097
Waist (cm)	−0.90 ± 3.32	−0.50 ± 4.15	0.493
Body fat (%)	−0.65 ± 1.16	−0.93 ± 4.17	0.529
Body fat (kg)	−0.34 ± 1.34	−0.52 ± 2.72	0.573
Lean body mass (kg)	1.09 ± 1.31	0.61 ± 1.24	0.008
Lean body mass (%)	1.93 ± 2.30	1.59 ± 2.99	0.380
App. skeletal muscle (kg)	0.67 ± 0.81	0.42 ± 0.62	0.016
App. skeletal muscle (%)	2.28 ± 2.76	2.07 ± 3.05	0.619
Quadriceps strength (N)	55 ± 57	52 ± 49	0.717
Quadriceps strength (%)	11 ± 14	14 ± 13	0.179
Grip strength (lb.)	3.2 ± 6.2	3.0 ± 5.4	0.784
Grip strength (%)	4.2 ± 8.5	6.7 ± 13.0	0.017
6MWD (m)	31 ± 38	35 ± 32	0.397
TUG (sec)	0.55 ± 0.98	0.71 ± 1.22	0.296
QoL (score)	0.64 ± 3.28	1.57 ± 3.85	0.077
MMSE (score)	0.41 ± 1.91	0.48 ± 1.83	0.817

^*∗*^Differences between genders according to independent samples *t*-test (normally distributed variables) and Mann–Whitney *U* test (not normally distributed variables).

**Table 4:** Logistic regression models showing the association between physical outcomes and the risk of drop-out.

Model 1					Model 2					Model 3				
Variable	Exp (*B*)	95% CI for EXP (*B*)		*P* value	Variables	Exp (*B*)	95% CI for EXP (*B*)		*P* value	Variables	Exp (*B*)	95% CI for EXP (*B*)		*P* value
6MWD (m)	0.991	0.986	0.995	<0.001	6MWD (m) Age (years)	0.994 1.102	0.988 1.013	1.000 1.198	0.048 0.024	6MWD (m)	0.994	0.988	1.000	0.063
										Age (years)	1.121	1.026	1.224	0.011
										Male	0.418	0.159	1.098	0.077

TUG (sec)	1.318	1.129	1.538	<0.001	TUG (sec) Age (years)	1.185 1.107	0.995 1.016	1.412 1.205	0.057 0.020	TUG (sec)	1.174	0.983	1.403	0.076
										Age (years)	1.128	1.032	1.234	0.008
										Male	0.394	0.141	1.095	0.074

Quad. strength (N)	0.993	0.988	0.998	0.006	Quad. strength (N) Age (years)	0.996 1.125	0.990 1.031	1.001 1.228	0.083 0.008	Quad. strength (N)	0.995	0.989	1.002	0.143
										Age (years)	1.123	1.018	1.239	0.021
										Male	1.062	0.286	3.949	0.928

Grip strength (lb.)	0.968	0.938	1.000	0.051	Grip strength (lb.) Age (years)	0.975 1.141	0.943 1.049	1.007 1.242	0.122 0.002	Grip strength (lb.)	0.960	0.913	1.010	0.116
										Age (years)	1.120	1.018	1.233	0.020
										Male	1.934	0.357	10.465	0.444

Exercise (min/week)	0.997	0.996	0.999	0.011	Exercise (min/week) Age (years)	0.998 1.126	0.996 1.048	1.000 1.210	0.032 0.001	Exercise (min/week)	0.998	0.995	1.000	0.029
										Age (years)	1.143	1.061	1.233	<0.001
										Male	0.349	0.137	0.891	0.028

Model 1 includes constant and physical measurement; model 2 includes additionally age; model 3: includes additionally gender; Exp (*B*) = hazard ratio.
